# Sclerotherapy of Vascular Malformations in the Oral Cavity—Minimizing Postoperative Morbidity

**DOI:** 10.3390/medicina56050254

**Published:** 2020-05-22

**Authors:** Itai Zeevi, Gavriel Chaushu, Michael Alterman, Liat Chaushu

**Affiliations:** 1 Department of Oral and Maxillofacial Surgery, Hadassah Hebrew University Medical Center, Jerusalem 91120, Israel; drzeevi@gmail.com (I.Z.); michael.alterman@gmail.com (M.A.); 2 Department of Oral and Maxillofacial Surgery, School of Dental Medicine, Tel Aviv University, Tel-Aviv 69978, Israel; gabi.chaushu@gmail.com; 3 Department of Oral and Maxillofacial Surgery, Rabin Medical Center, Campus Beilinson, Petah Tiqwa 49100, Israel; 4 Department of Periodontology and Implant Dentistry, School of Dental Medicine, Tel-Aviv University, Tel-Aviv 69978, Israel

**Keywords:** vascular malformation, sclerotherapy, ethanolamine oleate, morbidity

## Abstract

Vascular malformations (VMs) are a wide vascular or lymphatic group of lesions common on the head and neck. The objective of this study was to assess the efficacy and morbidity of sclerotherapy for the treatment of VMs in the oral and perioral area. Special attention was given to factors that may contribute to minimizing postoperative morbidity. Data from 25 patients (32 lesions) with oral VMs submitted to sclerotherapy with monoethanolamine oleate (EAO) were included. A structured form was used to collect data. An arbitrary score was determined to evaluate postoperative morbidity. Each of the following signs or symptoms received one point: pain, swelling, hematoma, ulceration, erythema, transient numbness, and transient itching. Pain and swelling were further divided into mild to moderate (1 point) and severe (2 points). Theoretically, the score was in the range of 0–9. Calculated scores ranged 0–4. The patients were further divided into two groups with scores of 0–1 denoting minimal morbidity (MIN) and 2–4 denoting significant morbidity (SIG). The number of lesions in each morbidity-score group were comparable (MIN 17and SIG 15). There were no statistically significant differences between the groups regarding age, number of applications, or average injection volume per mm lesion. Statistically significant differences were noted regarding gender (*p* = 0.05), lesion diameter (*p* = 0.030), total volume of first (*p =* 0.007) and second application (*p =* 0.05), and total injected volume (*p* = 0.03). Factors contributing to the risk for significant morbidity included being male, lesion diameter > 5 mm, volume > 0.3 mL per application, and total injected volume > 0.3 mL. A waiting time of 12 weeks prior to additional EAO application was required in 12 out of 29 lesions for clinical observation of complete regression. It was concluded that sclerotherapy with EAO as monotherapy is easy to apply, safe, and effective within a small number of sessions. Application of <0.3 mL EAO per session, and a waiting time of 12 weeks prior to the second application, would significantly minimize morbidity.

## 1. Introduction

Vascular malformations (VMs) are a wide vascular- or lymphatic-system heterogeneous group of lesions common in the head and neck [1]. Pathogenesis is associated with the disrupted morphogenesis of the endothelium (e.g., proliferation, migration, adhesion, differentiation, and survival) [2]. In order to promote standard communication between practitioners, in 1996 the International Society for the Study of Vascular Anomalies (ISSVA) classified vascular anomalies as “vascular tumors” (hemangiomas) and “vascular malformations” according to the pathology’s biological characteristics. Vascular malformations (VMs) were further classified as simple, combined, of major named vessels, associated with other anomalies, and high- or slow-flow lesions [3,4]. VMs can be divided into low-flow (venous, capillary, or lymphatic component) and high-flow (arterial or arteriovenous component) [5]. The majority of VMs in the oral cavity are venous and slow-flow in nature. The main affected areas are the lips, tongue, buccal mucosa, and palate. VMs in the oral cavity may lead to esthetic disorders, pain, and bleeding [6,7,8]. Spontaneous regression is rarely observed. Moreover, VMs can expand and are either single or multiple [8]. Signs and symptoms include pain, ulcerations, bleeding, discomfort, and cosmetic disturbance [6,9,10]. Trauma, pregnancy, or hormonal factors were implied as promoters [4]. Treatment is thought to be necessary in the presence of clinical symptoms, personal discomfort, or cosmetic disturbance. Different treatment modalities were proposed, including surgery, laser, embolization, cryotherapy, sclerotherapy, and corticosteroids [8,11,12].

Recent studies pointed out the potential role of mediators, e.g., vitamins and asymmetric dimethylarginine (ADMA), in vascular malformations [13,14]. Analysis of over 2100 developed drugs identified cholecalciferol (vitamin D3) as a potential therapeutic option [15].

Sclerotherapy is an efficient and conservative method for the treatment of VMs. It is a simple procedure that consists of intralesional injection with a low recurrence rate, good esthetic results, and reasonable morbidity [12]. Among the various available sclerosing agents, 5% monoethanolamine oleate (EAO) is characterized by high efficacy and a low toxic effect. The efficiency and safety of sclerotherapy using EAO for the treatment of VMs in various regions outside the oral cavity is well-established [1,16,17,18]. 

The objective of the present study was to assess efficacy and morbidity following sclerotherapy (intralesional injection of EAO as monotherapy) of VMs in the oral and perioral areas. Special attention was given to factors that may contribute to minimizing postoperative morbidity. The null hypothesis was that postoperative morbidity is dose-dependent. 

## 2. Materials and Methods

### 2.1. Characterization and Data Collection

This was a retrospective descriptive study based on data collection from patients with oral VMs submitted to sclerotherapy with EAO at the Department of Oral and Maxilofacial Surgery, Rabin Medical Center, Petah-Tikva, Israel. An electronic and manual database search was performed to locate patients diagnosed with VM in the oral cavity treated by sclerotherapy. The electronic search used the terms “vascular”, “vascular malformations” and “vascular lesions” from 2013 to 2017, resulting in 25 cases that were included in the present study. The hospital ethics committee approved the study in accordance with the Declaration of Helsinki (protocol no. 14-0190). 

A structured form was used to collect demographic data (age, gender), diagnostic resources used, location and size (greatest diameter) of the VMs, dosage and number of intralesional applications of EAO, interval between treatment sessions and follow-up sessions, intra- and postoperative complications, response to treatment, morbidity, patients’ satisfaction, recurrence, and total follow-up period. Morbidity was further classified as minor-to-moderate or severe according to intensity and duration of symptoms, patients’ return to the clinic, and the need for further treatment (medications or other).

### 2.2. Inclusion Criteria

- Clinical observation of painless purplish vesicles or bullae with soft consistency on palpation.
- Diascopy showing changes in coloring, intralesional ischemia, and decrease or alteration in e shape.

Lesions were diagnosed as low-flow VM on the basis of a patient’s anamnesis, and the clinical evaluation of the color, consistency, and size of the lesion. Diascopy (performed by applying pressure and observing color changes), the diagnostic injection of a vasoconstrictor agent (observing size and color changes), and aspiratory puncture and auscultation were also performed as needed.

### 2.3. Exclusion Criteria

- High-flow VMs,
- inadequate available data, and
- Patient’s medication interfering with wound healing (e.g., steroids, bisphosphonates, anticoagulants) or specific states preventing the use of EAO (e.g., pregnancy, lactation).

### 2.4. Sclerotherapy

**Figure 1:**
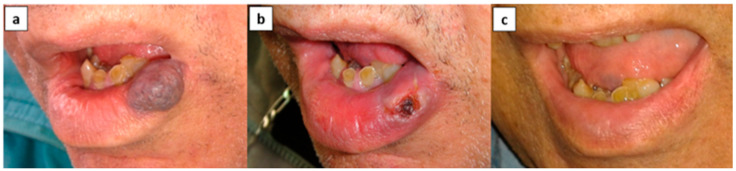
Patient no. 2. (**a**) Lower-lip vascular malformation (VM) before treatment, (**b**) ulceration following monoethanolamine oleate (EAO) sclerotherapy, and (**c**) complete resolution.

Application of the sclerosing solution followed manufacturer’s instructions Figure 1. Local anesthesia was provided using lidocaine 2% adrenalin 1:100,000 using a block technique away from the lesion to avoid access of the local anesthetic to the lesion. EAO was not diluted to allow maximal volume injection of the active EAO. The injection was applied in the central region of the VM with introduction of the needle to a depth that included half the volume of the VM. Before injecting the sclerosing solution, positive blood aspiration was verified. Blanching and/or progressive increase in the pressure for injection and/or leakage from the lesion surface were used as criteria for interrupting the procedure. 

### 2.5. Morbidity Score

An arbitrary score was determined to evaluate postoperative morbidity. Each of the following signs or symptoms received one point: pain, swelling, hematoma, ulceration, erythema, transient numbness, and transient itching. Pain and swelling were further divided into mild-to-moderate (1 point) and severe 2 (points). A morbidity score was calculated for each patient. Theoretically, the score could be in the range of 0–9. Calculated scores ranged 0–4. The patients were further divided into two groups, 0–1 for minimal morbidity (MIN) and 2–4 for significant morbidity (SIG). 

### 2.6. Statistical Analysis

Data were collected using a Microsoft Excel 2016 spreadsheet (Microsoft Corp, Redmond, WA, USA). Statistical analyses were performed using SPSS Statistics for Window and version 22.0 (IBM Corp, Armonk, NY, USA). Kolmogorov–Smirnov tests were conducted to test normal distribution. Measurements showed normal distribution (*p* > 0.05). Descriptive statistics were produced, and means (M) and standard deviations (SD) were calculated for all continuous measurements. Categorical data were expressed as fractions (number of occurrences divided by the total dataset), and fractions were also expressed as percentages. Statistical analyses of differences between the groups were assessed using Student’s *t*-test for continuous parameters and the chi-squared test for categorical data. *p* values lower than 5% were considered statistically significant.

## 3. Results

Twenty-five patients, 15 females (60%) and 10 males (40%), with a total of 32 VM lesions were included in the study. Table 1 summarizes the main demographic data and the clinical characteristics. Mean age was 56 ± 17 years. Fourteen patients were below the age of 60.

**Figure 2:**
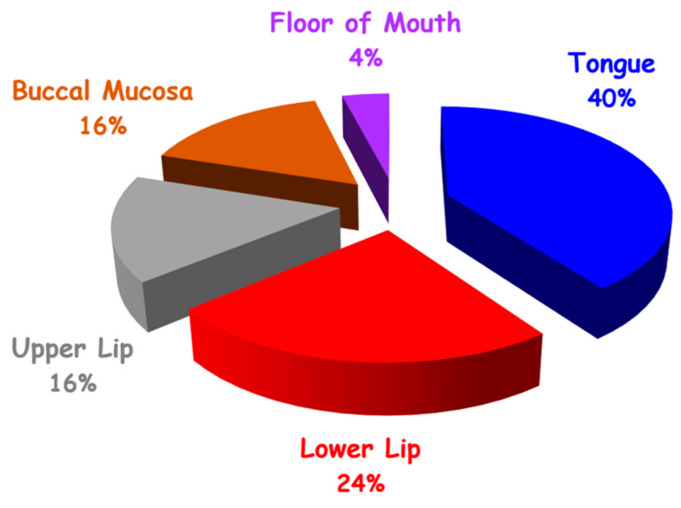
Distribution of VM in oral cavity by site (N = 32).

The most frequently involved area (Figure 2) was the tongue (13 lesions, 41%) followed by the lower lip (8 lesions, 25%), upper lip (5 lesions, 16%), buccal mucosa (5 lesions, 16%), and floor of mouth (1 lesion, 3%).

Lesion diameter was in the range of 3–35 mm (Figure 2). The main reason for intervention was esthetic disturbance (14 patients, 56%), followed by discomfort (9 patients, 36%), bleeding (7 patients, 28%), and pain (3 patients, 12%).

**Figure 3:**
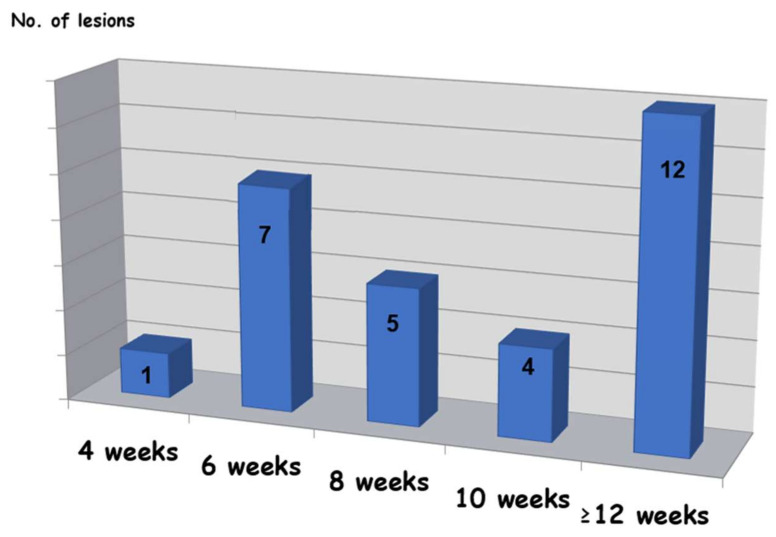
Time required for complete resolution.

The volume of injected EAO (Figure 3) ranged from 0.1 to 4 mL with an average of 0.06 mL per 1 mm of lesion diameter.

The majority of lesions were treated by a single application of EAO (25, 78%), while 6 (19%) underwent two treatment sessions and one case (3%) needed three treatment sessions. Among the seven cases that underwent more than one treatment, four sessions were held within a 1–2 week interval from the previous session. Follow-up sessions were conducted with a 1–3 week interval and until a total resolution of the lesion was observed. 

Twenty-nine lesions (~91%) showed total clinical regression (Figure 3), two lesions only achieved partial resolution, and 1 could not be determined because the patient was lost from the follow-up sessions. Among two cases with partial response, the outcome was sufficient for patients’ satisfaction because symptoms of pain, discomfort, and esthetics were improved, and they did not want to continue with the treatment.

In most cases (28/29 lesions, 97%), healing was still in process after 1–3 weeks, and the response for treatment could not be determined at that time. In 97% of the cases (28/29 lesions), more than dix weeks were needed for complete resolution, while in 72% (21/29 lesions), complete healing was observed after eight weeks or more. In 41% of the cases (12/29 lesions), the required waiting time to resolution was 12 weeks.

**Figure 4:**
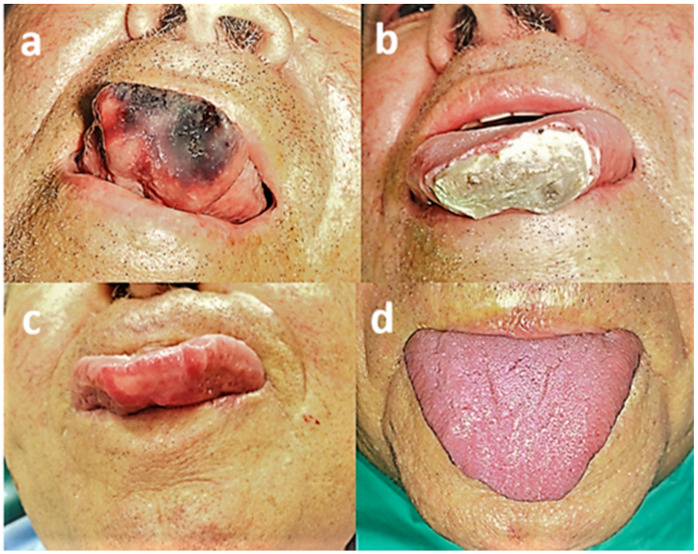
Patient no. 8. (**a**) Clinical view prior to treatment; (**b**) necrotic tissue on anterior tongue part following sclerotherapy; (**c**) clinical view following debridement; (**d**) complete healing.

Most of the patients (19, 76%) experienced side effects to some degree. Common side effects included pain (11, 44%), swelling (7, 28%), hematoma (6, 24%), and ulceration (5, 20%). Less common side effects included erythema (2, 8%), transient numbness of the lower lip (1, 4%), and transient itching sensation of the lower lip (1, 4%). One patient developed necrotic tissue on the anterior part of the tongue (Figure 4), requiring intervention for debridement, after which complete healing was achieved.

The number of lesions in each morbidity-score group were close (MIN-17; SIG-15). There were no statistically significant differences between groups regarding age, number of applications, or average volume per mm lesion (Table 2). Statistically significant differences were noted regarding gender (*p =* 0.05), lesion diameter (*p* = 0.030), volume of first application (*p* = 0.007), volume of second application (*p =* 0.05), and total injected volume (*p =* 0.03). Patients of male gender with lesion diameter > 5 mm, volume > 0.3 mL per application, and total volume > 0.3 mL showed higher morbidity.

## 4. Discussion

The subjects included in the present study were adults with a mean age of 59 ± 18 years. Other studies reported a higher prevalence of VMs in the young [19,20,21].

Sixty-one percent of VMs were in women, as reported in other studies [8,21]. However, in the MIN group, women’s prevalence was 76%, while in the SIG group, there was male predominance (53%). Increased morbidity in males was reported in this study for the first time. Future studies are required to further investigate this correlation.

VMs were most frequently found on the tongue, whereas in most of the literature, the lower lip is the most frequent site [6,7,20]. One explanation may be the performance of this study in a maxillofacial department. Patients with lip lesions approach plastic surgeons or dermatologists more frequently than patients with lesions in other areas. 

All the VMs were venous with slow blood flow. Success rate in such cases is in the range of 70–100% [1,20,22,23,24,25,26,27,28]. It was previously reported that, for smaller lesions, there is no need for complementary therapies [22]. In the present study, lesions ranging up to 35 mm did not require any additional therapy. Consequently, it could be deduced that sclerotherapy with EAO is effective with low toxicity [7,16]. In addition, postoperative morbidity is minimal following less invasive than surgery [1,28]. 

Ideal EAO concentration and dosing for treatment is still debatable [1,8,19,20]. A previous suggestion was <1 mL per session [29]. Hyodoh et al. (2005) reported that a single application was sufficient for total resolution in smaller vascular lesions (≤30 mm) [17]. On average, 1.25 sessions were needed for resolution, 1.29 in the MIN group and 1.2 in the SIG group. Results are comparable to those in previous studies [19,20,28]. We suggest that a 5% concentration is effective compared to an average of 3.7 sessions with concentrations of 1.5% and 2.5% as reported by Johann (2005) [1].

Complications in sclerotherapy are dose-dependent [19,30,31,32]. Excessive EAO amount or high pressure may be responsible [28,32]. In the present study, light pressure and positive aspiration were used to minimize morbidity. Patients undergoing hematopoietic stem-cell transplantation receive high doses of chemotherapy and radiotherapy that cause severe immunosuppression. Mucositis accounts for an increased morbidity score and the potential risk of clinical complications [33,34,35]. These complications resemble those seen in the present study. The morbidity score in the present study enabled assessment of additional factors contributing to morbidity. Unlike total injection volume, gender and lesion diameter were not under operator control. The total morbidity of both groups was not high. However, to further minimize morbidity without endangering efficacy, it is suggested that a volume not exceeding 0.3 mL should be applied per session. A longer waiting time between sessions is also suggested. 

The present study demonstrated that, in most cases (72%), complete healing was observed after 8–12 weeks, while in 41% of the cases, the required waiting time to resolution was 12 weeks. Consequently, no additional applications were required. In the present study, the required interval between treatment sessions and the waiting time necessary for complete healing were longer than previously reported [20]. A total application volume not exceeding 0.3 mL was associated with increased morbidity. Therefore, one application of 0.3 mL EAO might be sufficient in most cases, provided there is also a longer waiting time between sessions.

There are no standard treatment algorithms available for VMs, and the disorder has significant unmet clinical needs. Improved understanding of the pathogenesis of vascular anomalies may provide insights to the development of new targeted therapies [2]. Recently, two factors emerged associated with epithelial dysfunction [13,14]. Vitamin D and asymmetric dimethylarginine (ADMA) play a crucial role in endothelial function, and may be links for the known interaction of chronic periodontitis (CP) and coronary heart disease (CHD). Patients with CP and CP + CHD had significantly lower serum levels of vitamin D compared to those with CHD and healthy controls. Moreover, the presence of CP negatively influenced serum vitamin D levels [13]. Patients with CHD and CP + CHD had higher levels of salivary and serum ADMA compared to healthy subjects and CP patients [14]. Future therapies targeted for increasing vitamin D [15] or decreasing ADMA might be alternatives for the medical treatment of VMs. 

Taking into consideration the limitations of the study, such as the reduced number of samples (32 lesions) and the fact that it was a retrospective study, the application of EAO as a monotherapy was an easy, simple, and quick treatment because it was performed as an outpatient procedure with a limited number of sessions. It was well-tolerated by patients and resulted in low morbidity. The data showed that this is an effective treatment in 100% of the cases without the need for surgery (Figure 1).

## 5. Conclusions

Sclerotherapy with EAO as a monotherapy is easy to apply, safe, and effective within a small number of sessions. Application of <0.3 mL EAO per session with a waiting time of 8–12 weeks before the second application significantly minimizes morbidity.

## Figures and Tables

**Table 1:** Patient data.

Patient	Lesion	Gender	Age (Years)	Location	Diameter (mm)	Indication	Application			Total Dose (mL)		Average Dose (mL/mm)	Score	Adverse Effects
							First	Second	Third	No.				
1	1	Female	42	Lower lip	5	Esthetics	0.5	0.25	0.25	3	1	0.200	1	Erythema
2	2	Male	69	Lower lip	30	Esthetics	1	1		2	2	0.067	3	Severe pain, ulceration
3	3	Male	57	Tongue	20	Bleeding	1	0.5		2	1.5	0.075	2	Severe pain
4	4	Male	81	Lower lip	30	Esthetics	1			1	1	0.033	3	Severe pain, ulceration
5	5	Female	72	Buccal Mucosa	15	Bleeding	2.5	1.5		2	4	0.267	3	Severe pain, hematoma
6	6	Female	61	Tongue	7	Esthetics, pain	0.5			1	0.5	0.071	0	
7	7	Male	61	Buccal Mucosa	10	Bleeding	2			1	2	0.200	3	Hematoma, severe pain
8	8	Male	51	Tongue	15	Bleeding	1.5			1	2	0.100	4	Severe pain, hematoma, necrosis
9	9	Female	73	Tongue	10	Discomfort	0.15			1	0.15	0.015	1	Mild pain
10	10	Female	53	Lower lip	20	Esthetics	0.75			1	0.75	0.038	3	Severe pain, mild swelling, transient numbness
11	11	Male	57	Upper lip	15	Esthetics	0.2			1	0.2	0.013	2	Hematoma, mild swelling
12	12	Female	64	Tongue	5	Discomfort	0.3			1	0.3	0.060	0	
12	13	Female	64	Tongue	8		0.3			1	0.3	0.038	0	
13	14	Female	43	Floor of mouth	8	Discomfort	0.5			1	0.5	0.063	0	
14	15	Male	23	Tongue	3	Bleeding	0.2	0.2		2	0.4	0.133	0	
14	16	Male	23	Tongue	30	Pain	0.5	0.5		2	1	0.033	0	
15	17	Female	56	Tongue	10	Discomfort	0.4			1	0.4	0.040	2	Ulceration, mild pain
16	18	Female	63	Lower lip	5	Esthetics, bleeding	0.3			1	0.3	0.060	2	Mild swelling, erythema
17	19	Male	54	Tongue	25	Discomfort	0.5			1	0.5	0.020	3	Severe pain, ulceration
18	20	Female	85	Lower lip	5	Esthetics	0.3			1	0.3	0.060	2	Ulcer, itching sensation
19	21	Male	79	Upper lip	5	Esthetics	0.3			1	0.3	0.060	0	
19	22	Male	79	Upper lip	3	Esthetics	0.2			1	0.2	0.067	0	
19	23	Male	79	Lower lip	7	Esthetics	0.4			1	0.4	0.057	0	
20	24	Female	78	Buccal Mucosa	5	Discomfort	0.3			1	0.3	0.060	2	Mild swelling, hematoma
20	25	Female	78	Buccal Mucosa	5	Discomfort	0.3			1	0.3	0.060	0	
20	26	Female	78	Buccal Mucosa	5	Discomfort	0.3			1	0.3	0.060	0	
21	27	Female	54	Tongue	15	Pain, bleeding	0.3			1	0.3	0.020	2	Mild swelling, mild pain
21	28	Female	54	Tongue	35		0.4	0.4		2	0.8	0.023	0	
22	29	Female	26	Upper lip	5	Esthetics	0.2			1	0.2	0.040	1	Mild swelling
23	30	Male	31	Lower lip	20	Esthetics	0.3			1	0.3	0.015	3	Severe swelling and hematoma
24	31	Female	29	Upper lip	5	Esthetics	0.1			1	0.1	0.020	0	
25	32	Female	41	Tongue	10	Discomfort	0.4			1	0.4	0.040	0	Mild pain

**Table 2:** Morbidity score. MIN, minimal morbidity; SIG, significant morbidity.

	Females (%)	Age (Years)	Diameter (mm)	First Application (mL)	Second Application (mL)	No. of Applications	Total Dosage (mL)	Average Dosage (mL/mm)	
Average	76	55	9	0.3	0.3	1.3	0.4	0.06	MIN
SD		21	9	0.1	0.1	0.6	0.2	0.04	
Median		61	5	0.3	0.3	1	0.3	0.06	
Minimum		23	3	0.1	0.2	1	0.1	0.015	
Maximum		79	35	0.5	0.5	3	1	0.2	
Average	47	61	16	0.8		1.2	1	0.07	SIG
SD		14	8	0.7		0.4	1	0.07	
Median		57	15	0.5		1	0.5	0.06	
Minimum		31	5	0.2		1	0.2	0.01	
Maximum		85	30	2.5		2	4	0.27	
*p* value	0.05	0.33	0.03	0.007	0.05	0.6	0.03	0.6	
